# SinoPedia—A Linked Data Services platform for decentralized knowledge base

**DOI:** 10.1371/journal.pone.0219992

**Published:** 2019-08-02

**Authors:** Tao Chen, Yongjuan Zhang, Zhengjun Wang, Dongsheng Wang, Hui Li, Wei Liu

**Affiliations:** 1 Shanghai Library/Institute of Scientific & Technical Information of Shanghai, Shanghai, China; 2 School of Information Management, Nanjing University, Nanjing, China; 3 Department of Library, Information & Archives Shanghai University, Shanghai, China; 4 Shanghai Information Center for Life Sciences, Chinese Academy of Sciences, Shanghai, China; 5 Department of Chemistry Physics, Lund University, Lund, Sweden; 6 Computer Science, University of Copenhagen, Copenhagen, Denmark; Xiamen University, CHINA

## Abstract

Knowledge bases are largely developed and utilized in academic and industrial fields, such as DBPedia, VIAF, LoC, Getty, and which are published in the Linked Data format. However, if you want to view different resources on these knowledge bases, you have to switch between different web pages from these resources. Therefore, we proposed a decentralized data hub named SinoPedia, which consists of several linked data services and can re-publish these RDF data in one platform. Firstly, these different Linked Data services include: Linked Data Transformation Service (LDTS), Linked Data Query Service (LDQS), Linked Data Publishing Service (LDPS) and Linked Data Knowledge Service (LDKS). The resource URI is the basis and core of the linked data, thus we will focus on the resource forwarding mechanism in LDPS service which can rewrite resource URI using a global and standard format among knowledge bases. Some knowledge bases were configured in SinoPedia platform in this paper. In addition to the above services, Linked Data Reasoning Service (LDRS) and Linked Data Intelligence Service (LDIS) will be added to the platform in the future. In short, all of these Linked Data Services will form the core framework in order to providing a good linked data application ecosystem.

## Introduction

The focus of Linked Data is about using the Web to connect related data that wasn't linked previously, or using the Web to lower the barriers to link the data currently linked by other methods[1–3]. A huge amount of Linked Data has been published in recent years and been ready for consumption in recent years. In the latest LOD (Linked Open Data) cloud, it contains 1234 datasets of 16136 links from life science to government yield. Some datasets can be accessed via SPARQL endpoints, however, other datasets can only be downloaded. At the same time, the SPARQL endpoint can be seen as a way of data publishing, whatever, this method of database retrieval is not convenient for resource circulation and sharing. For example, if you want to query another resources information related to the resource, you must re-execute SPARQL. There are some knowledge bases providing online access to browse their metadata, however, different dataset resources need to be browsed on different websites, which brings confusion and inconvenience to end users.

In recent years, more and more institutions and scholars are studying the integration of data in different datasets, such as building a multi-database datahub.

LDF(Linked Data Fragment) is a publishing method that allows efficient offloading of query execution from servers to clients using a lightweight partitioning strategy. This method enables servers to maintain availability rates as high as any regular HTTP server, allowing querying to scale reliably to much larger numbers of clients. LDF proposes an efficient storage mechanism, and suitable for browsing data inside a dataset[4–5].

KBpedia is a comprehensive knowledge structure for promoting data interoperability and knowledge-based artificial intelligence. The structure of KBpedia knowledge combines seven "core" public knowledge bases—Wikipedia, Wikidata, schema.org, DBpedia, GeoNames, OpenCyc, and UMBEL. This method is more biased towards data applications, rather than the presentation of metadata[6].

OpenKG.CN and datahub.io sites have a large amount of open datasets, and they are used more as a content management platform that provides data download and linking to the dataset's original address of the dataset. In addition, the methods can't provide online unified viewing of the contents of the original dataset.

KaBOB is an ontology-based semantic integration of biomedical databases which includes 14 ontologies and large RDF data converted from 18 data sources in bioinformatics[7]. Bio2RDF is a method which has successfully applied the semantic web technology to publicly available databases using creating a knowledge space of RDF documents linked together with normalized URIs and sharing a common ontology[8–9]. Thus, the datasets should be imported and stored in these two platforms.

Data publishing is the basis of linked data applications, and it needs to follow the four principles provided by Berners-Lee in 2006. Considering that the publishing process has technical thresholds, we propose the SinoPedia platform. This platform can be used as a unified publishing platform to publish RDF data of different distributed datasets with simple configuration. We summarize the main contributions of this work as follows:

1. We encapsulate a large number of semantic web technologies in SinoPedia platform so that there can be easily applied in semantic applications through simple service calls.
2. We propose a property alignment method which can be used to map SinoPedia classes and properties to DBPedia core ontology.
3. We design and perform a URI forwarding mechanism that preserves the original rules and states of resources to the maximum extent possible, while also making it easy to distinguish resources between different endpoints in SinoPedia platform.

This paper is structured as follows: in the next section, we describe the framework of SinoPedia knowledge base, a linked data service platform we developed, which contains LDTS, LDQS, LDPS, and LDKS services. In Section 3, we provide a property alignment method to show how to select the properties of DBpedia knowledge base in SinoPedia. Moreover, we describe how to define resource URIs between multiple SPARQL endpoints in Section 4. In Section 5, we demonstrate some cases to explain how to apply the linked data services provided by SionPedia in applications. We conclude the result in Section 6.

## SinoPedia platform (LDSP) framework

**Fig 1:**
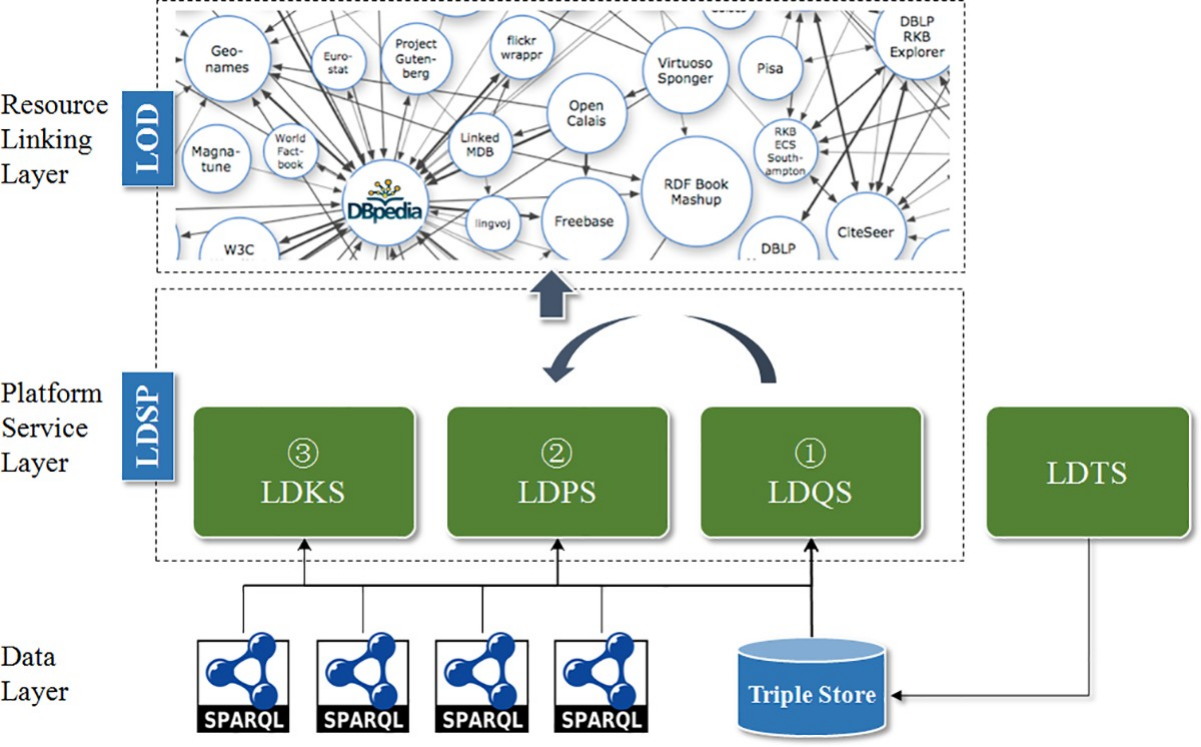
SinoPedia: Linked data service platform framework.

As shown in Fig 1, SinoPedia platform framework is divided into three layers: data layer, platform service layer and resource linking layer[10].

1. Data Layer

This layer is the persistence storage layer for whole platform which includes two types of data sources.

**Private Data**: Private data, generated by public customers with LDTS (Linked Data Transformation Service), will be stored in triplestore database (OpenLink Virtuoso). For LDTS service, there are several methods to convert data to RDF format, such as D2R[11] and R2RML[12] are the two common ways to convert relational database data to RDF; and OpenRefine tool can be applied to generate RDF data for excel files, etc.

Because the structured, semi-structured, and unstructured data can be transformed to RDF offline using various technologies and tools, we don't describe LDTS more in paper.

**Remote SPARQL Endpoints**: A large number of knowledge bases provide endpoint access interfaces, thus we can retrieve data from these endpoints online. Remote public knowledge bases are bound to SinoPedia platform using system configuration file. This will be discussed in section 4.

2. Platform Service Layer

The core layer of the SinoPedia platform consists of four service features.

**Linked Data Query Service (LDQS)**: SinoPedia provides LDQS service, which can be used to query data in different SPARQL endpoint with web service interface. Federated query can also be executed in SPARQL endpoint, but the performance is not good in practice. In the future, we will use the LDF Server instead of the current query method.

**Linked Data Publishing Service (LDPS)**: This service can be used to publish RDF data of local and remote SPARQL endpoints. We made secondary development based on LODVIEW software[13], extended the endpoint connection function, and enabled the system to support multi-endpoints configuration. LODVIEW is a Java web application based on Spring and Jena. This tool can offer a W3C standard compliant IRI dereferenciation. It also allows user to publish RDF data according to all defined standards for Linked Open Data in conjunction with a SPARQL Endpoint.

**Linked Data Knowledge Service (LDKS)**: The relationship of resource among multiple SPARQL endpoints will be shown in knowledge graph. This service mainly integrates LODLIVE software[14], which is an experimental project that was setup to spread and promote the linked open data philosophy and to create a tool that can be used for connecting RDF browser capabilities with the effectiveness of data graph representation.

3. Resource Linking Layer

Traditionally, with linked data, when you have some of it, you can find other from the related data[15]. There are many technologies to achieve connection of resources, NLP, machine learning, entity disambiguation, etc. There are also other useful tools integrated into these technologies and there are conveniently used to connect resources among multiple knowledge bases, such as LDIF (Linked Data Integration Framework), SILK, LIMES[16], etc.

## Property alignment in LDTS

During the process of conversion in LDTS service, the properties have been aligned among them and with standard existing ontologies to achieve conceptual interoperability. Moreover, several refinement steps have been performed to conform to international standards. In the subsection we will give some more details about the alignment and refinement processes. As the one of the largest knowledge base of linked data application, the full DBpedia dataset features 38 million labels and abstracts in 125 different languages, 25.2 million links to images and 29.8 million links to external web pages; 80.9 million links to Wikipedia categories, and 41.2 million links to YAGO categories. In addition, DBpedia is connected with other Linked Datasets by around 50 million RDF links approximately. Thus, we align SinoPedia to DBPedia core ontology, and you can expand vocabularies if these properties cannot meet the data structure needs[17].

As a common encyclopedia website, everyone can edit and contribute their knowledge. Therefore, the customized edition and the diverse vocabularies lead to an extraordinary amount of properties in infobox. For instance, there are tens of thousands of properties in DBPedia. Moreover, we do not necessarily adopt all of them, and nor organize them as synsets. According to our partially statistical data, less than one out of eight takes up 90% of all instances; while the other seven out of eight properties, only covers 10% of instances. Many of the properties only have one or two instances, indicating they are strongly customized habits of editing. Selecting a suitable number of properties for reuse in SinoPedia platform will maintain the majority coverage of all instances. Simultaneously, it could increase the quality of knowledge base.

Hence, we classify the properties for each top category according to their appearance in actual instances. It is noted that the same property could appear in different instances belonging to distinct top categories, resulting in different property domains (top categories), for example, "name", "belongTo" could be employed in different domains.

Then, we have to drop the outlier properties, since most of which only appear once (in one instance) due to diverse and informal habits of users. To balance the amount of properties and maintain a high coverage of instances, there are three concerned factors:

1. Drop as many properties as possible; record it as the dropping ratio, which should be the higher, the better.
2. The properties left still cover a high percentage of the instances number.
3. We rank the instance numbers with respect to properties in advance, and always drop the properties with the least instances in sequence.

Therefore, we normalize the factors as Eqs (1) and (2) for the set of dropped properties D in the following equations:

$$score(D)=\frac{\left|D\right|}{\left|T\right|}\times \frac{\sum_{p\in (T-D)}\left|Instance_{p}\right|}{\left|Instance\right|}$$ (1)

$${select}_{D}(T)={argmax}_{D∁T}score(D)$$ (2)

where $\frac{\left|D\right|}{\left|T\right|}$ is the dropping ratio. |D| is the number of dropped properties, and |T| is the total number of all properties. $\frac{\sum_{p\in (T-D)}\left|Instance_{p}\right|}{\left|Instance\right|}$ is the ratio of instance coverage for the remaining properties. *p* is a property from the remaining set (*T−D*), and |*Instance_p_*| is the total instance number concerned with the property *p*. With the increase of |D|, the coverage of aggregated instances for the remaining (T-D) would reduce. Therefore, we select D with the greatest value of *score*(*D*) of their product.

**Fig 2:**
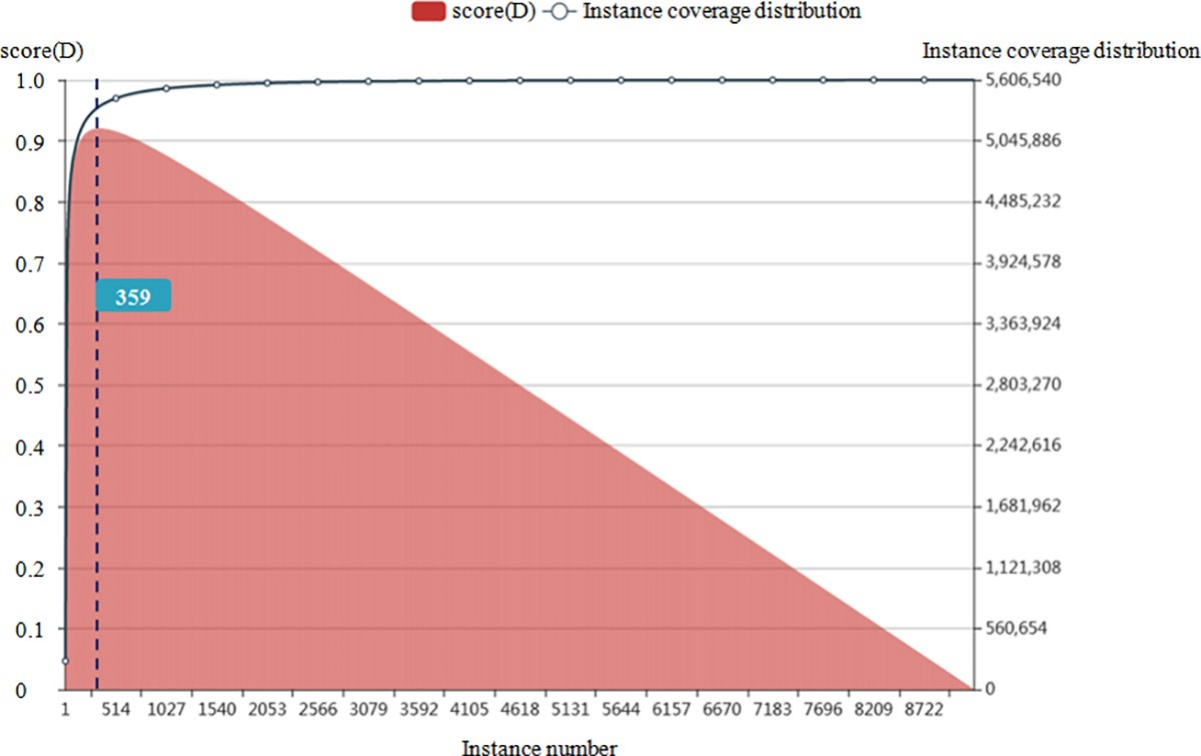
Score(D) and instance coverage distribution for different size of |T-D| for person category.

Currently, we choose several classes in SinoPedia, person, organization, place and temporal. Moreover, there are 1818072 instances in Person class in DBPedia, and for Person class, it has 9230 properties (T = 9230). Therefore, we should confirm how many properties should be selected in SinoPedia in order to keep the majority coverage of all instances. We know one instance have multiple properties, and the same property can be used in multiple instances. Therefore, we use instance occurrence number as total count. We can see that the score(D) will be declined from 360 line, as shown in Fig 2 and Table 1. We can reuse 359 properties from Person class in DBPedia knowledge base in SinoPedia platform. In this table, column "value" is the cumulative quantity with the increase of |T-D|.

**Table 1:** Properties list for person category in DBPediaknowledge base.

*No.*	*Property*	*{D}*	*{Value}*	*Score(D)*
1	rdf:type	9 229	261 561	0.0466477312
2	foaf:name	9 228	516 044	0.0920231789
3	rdfs:label	9 227	730 439	0.1302408874
. . . ..				
359	dbo:sopt	8 871	5 370 235	0.9205954075
360	dbp:startyear	8 870	5 370 840	0.9205953479
. . . . .				
9230	dbp:players	0	560 6540	0

## URI design in LDPS

Uniform Resource Identifiers (URI), which is a key technology to support Linked Data by offering a generic mechanism to identify entities ("Things") or concepts in the world, is a single global identification system used on the World Wide Web. Every resource is identified using a URI for linked data knowledge bases. URI has been designed with simplicity, stability, traceability and manageability principles in mind. We must maintain consistency with the original resources in LDPS service so that to ensure the "uniqueness" of connected URIs. Therefore, for designing URIs, we don't change the original resource URI, only forwarding them with new URI. The URI format is:

http://{**ldps_domain**}/${**endpoint_label**}/${**resource_pattern**}.

ldps_domain is the host of SionPedia platform. The endpoint_label is the tag used to distinguish different data sources. resource_pattern represents original resource pattern.

**Fig 3:**
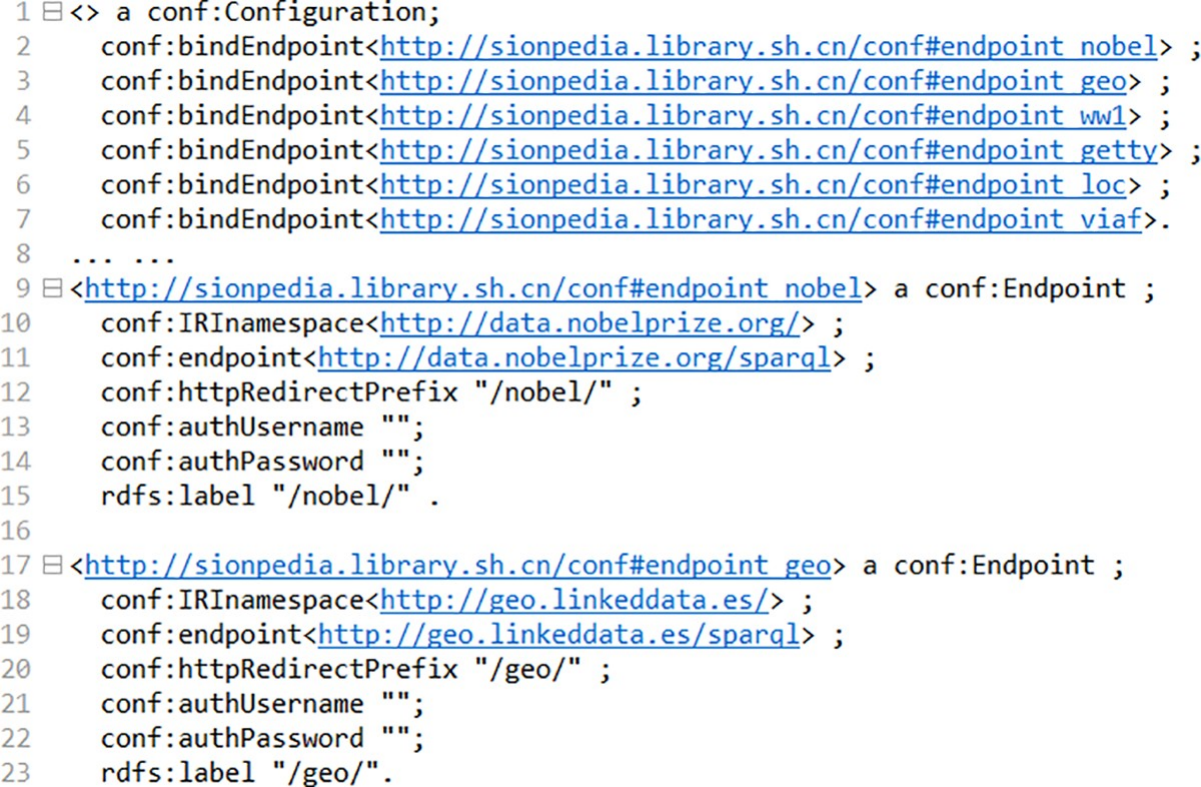
Code for configuration file.

Firstly, it has been shown that how to bind and identify different resource endpoints in SinoPedia. The following code in Fig 3 shows an example of two connected nodes, endpoint_nobel and endpoint_geowhich are the two remote datasets with SPARQL endpoint "http://data.nobelprize.org/sparql" and "http://geo.linkeddta.es/sparql". In order to distinguish different knowledge base nodes, we use rdfs:label property to tag them ("/**nobel**/" and "/**geo**/").

**Fig 4:**
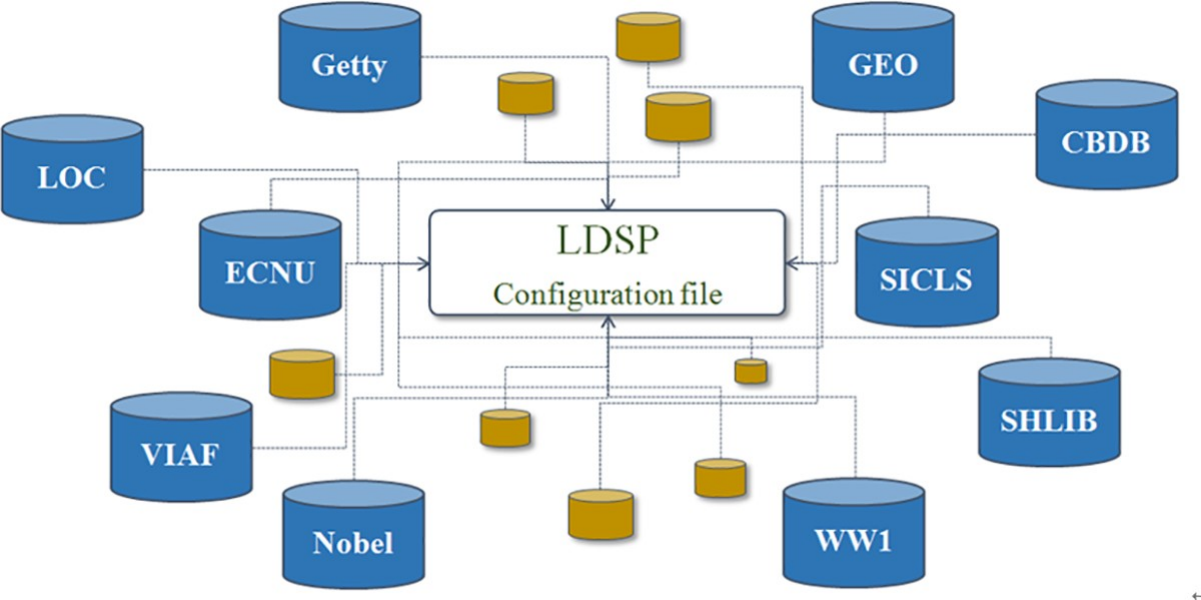
Connecting SPARQL endpoints with configuration file in LDSP.

With this configuration file, the multiple knowledge bases can be linked to SinoPedia as shown in Fig 4. We have bounded the dataset including Getty, GEO, CBDB, LoC, ECNU (East China Normal University)—chorography works, SICLS (Shanghai Information Center for Life Sciences)—article data, SHLIB (Shanghai Library)—digital humanities projects, VIAF, NobelPrize and WW1.

Here, we give same examples to explain how to re-publish remote resource URIs in LDSP of SinoPedia. Such as, Tsung-Dao (T.D.) Lee, a resource from nobelprize knowledge base (SPARQL endpoint: http://data/nobelprize.org/sparql). Lee's URI is the link of "http://data.nobelprize.org/resource/laureate/69" where the pattern part is "resource/laureate/69". In SinoPedia, we have set the endpoint label to "nobel" for this knowledge base. As a result, we can use the new SinoPedia URI (S-URI) to require the metadata of resource. In the same way, other resources can also be rewritten the S-URI. In Table 2, the resource "1st Army" S-URI is the link of "http://sinopedia.library.sh.cn/ww1/ww1lod/635e1f7d" which is extracted from SPARQL endpoint "http://ld.fi/ww1lod/sparql".

**Table 2:** Examples for resources URI forwarding.

Resource	Original Resource	Endpoint Label	SinoPedia URI
Tsung-Dao (T.D.) Lee	http://data.nobelprize.org/*resource/laureate/69*	Nobel	http://sinopedia.library.sh.cn/nobel/*resource/laureate/69*
1st Army	http://ldf.fi/*ww1lod/635e1f7d*	ww1	http://sinopedia.library.sh.cn/ww1/*ww1lod/635e1f7d*
Diamond Brook	http://vocab.getty.edu/*tgn/1135223*	Getty	http://sinopedia.library.sh.cn/getty/*tgn/1135223*
Sein, Lago del	http://geo.linkeddata.es/*resource/Lago/Sein%2C%20Lago%20del*	geo	http://sinopedia.library.sh.cn/geo/*resource/Lago/Sein%2C%20Lago%20del*

**Fig 5:**
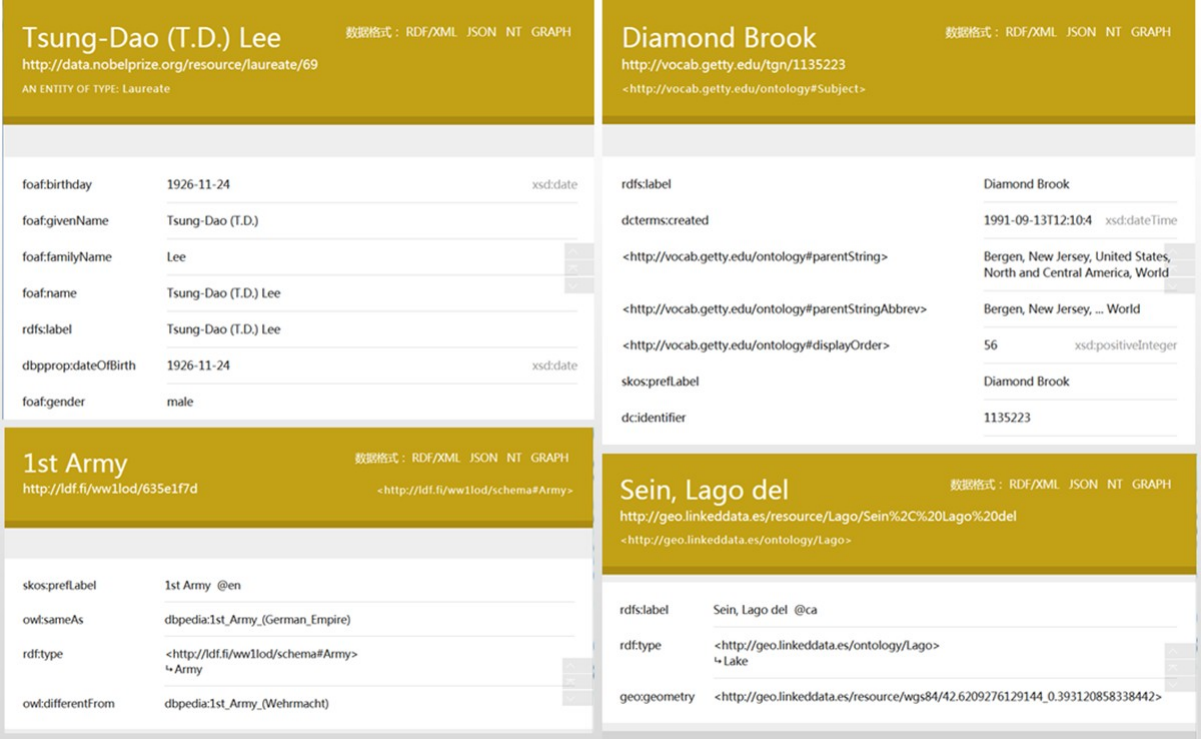
Publishing multiple knowledge bases in SinoPedia platform.

The results of the four resources in SinoPedia are shown in Fig 5. Different resources in multiple datasets are browsed in the same format in SinoPedia.

## Experiments and results

The SinoPedia dataset consists of millions of triples generated by LDTS service. This dataset can be queried by selecting the RDF graph named "http://sinopedia.library.sh.cn/graph/shlib" on the dedicated SPARQL endpoint from the link of "http://sionpedia.library.sh.cn/sparql". Currently, the SinoPedia contains 684175 instances of Person category, 30351 instances of Organization category and 3972 instances of Place. These instances are also linked to some public knowledge bases, for example, Person instances are linking to DBpedia, Shanghai Library Public Data, CBDB, and Nobelprize endpoints.

All triples, private data and connected remote endpoints are also accessible through content negotiation which is a flexible point in the Web architecture. The goal is to develop the Web by introducing different formats. Such as:

"{resource_uri}.rdf" will response rdf/xml format.
"{resource_uri}.json" will response json_ld format.
"{resource_uri}.ntriples" will response ntriples format.

**Fig 6:**
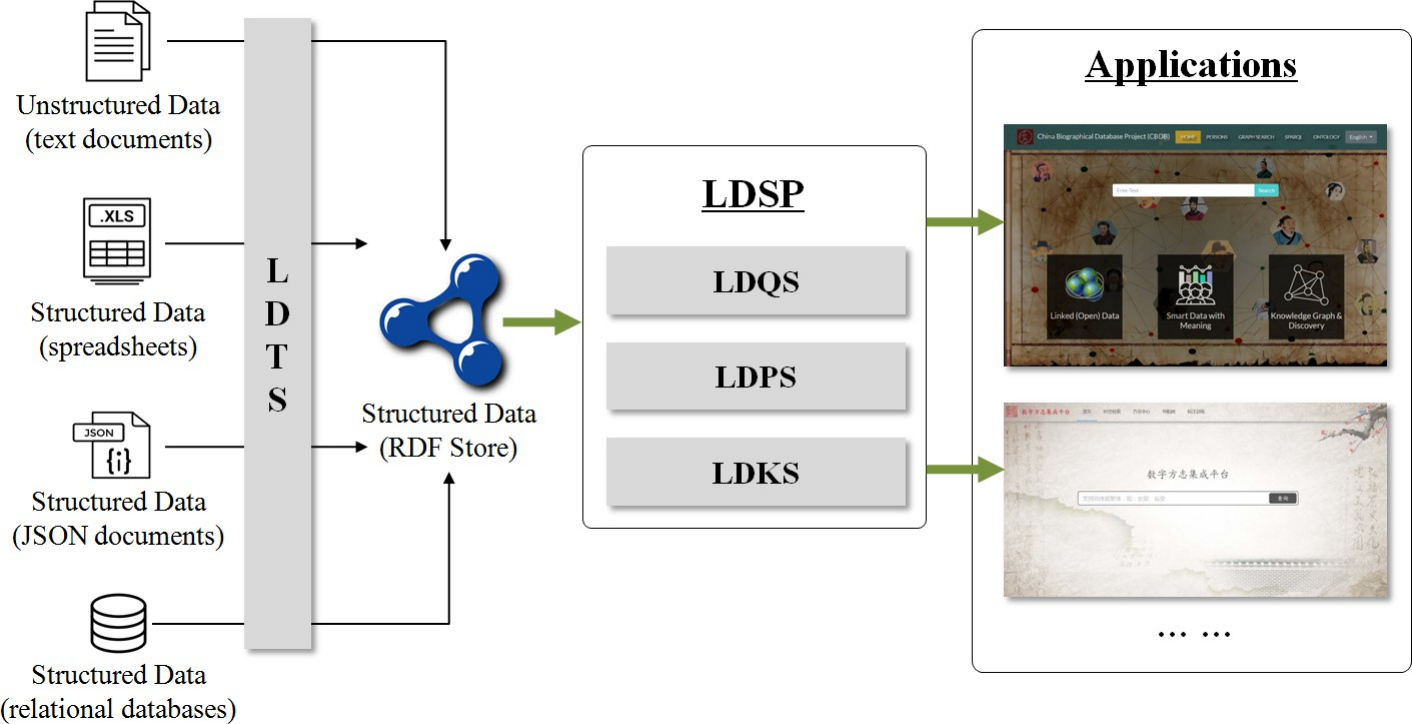
Apply linked data services in applications.

Fig 6 shows the workflow about how to use linked data services in applications. We have applied the services provided by the LDPS platform in several projects, and introduce two representative projects in this section.

### Case 1: The China Biographical Database (CBDB) Linked Data Platform

The China Biographical Database (CBDB) is a free accessible relational database with biographical information about approximately 422600 individuals, primarily from the 7th to 19th centuries. The original CBDB system provides the API access to retrieve data in JSON format. We developed a linked data version (CBDB_LD, http://cbdb.library.sh.cn) for CBDB dataset released at SILF 2018 (Shanghai Library Forum 2018), aiming at organizing and publishing data in RDF format and linking them with external linked open data to merge more knowledge.

LDTS service can be completed offline, therefore, we use Python program to crawler the JSON data with CBDB API, and transform these data to RDF format which are stored in OpenLink Virtuoso database (RDF Store) in this application. LDQS service provides a form of interface acquired by the machine, similar to the API interface. This service is closely related to each project. Thus, we will not introduce this part in detail.

Next, we discuss how to call LDPS service and LDKS service in applications.

#### 1. LDPS service

After LDTS, all data is already in RDF format which are published on SionPedia platform with the open standards recommended by the W3C through a simple configuration. In publishing, SinoPedia platform provides multiple serialization formats for RDF data, such as RDF/HTML, RDF/XML, JSONLD, NT. These serialization formatted data can be retrieved via URL addresses, thus, there can be easily called remotely in CBDB_LD project.

**Fig 7:**
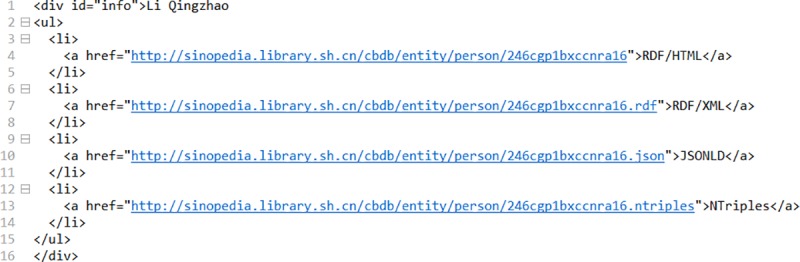
Code for LDPS service in a program.

**Fig 8:**
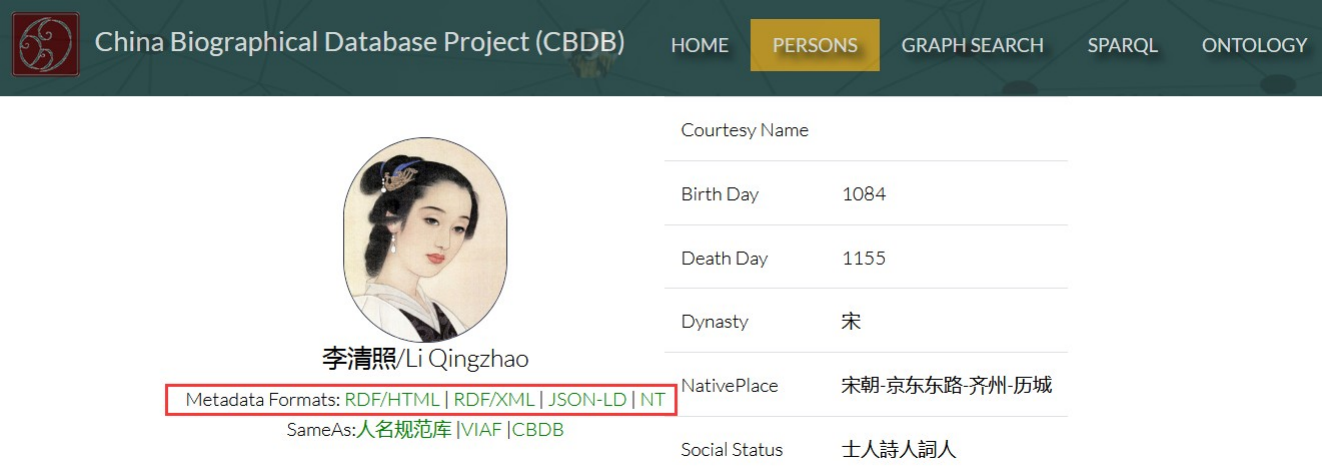
LDPS service in screenshot for "Li Qingzhao".

Here we give some examples to show how to call LDPS service. In RDF store, the resource URI of "Li Qingzhao" is "http://cbdb.library.sh.cn /entity/person/246cgp1bxccnra16", and the redirected published URI of this resource is changed to "http://sinopedia.library.sh.cn/cbdb/entity/person/246cgp1bxccnra16". In this new URI, we can see the domain is "sinopedia.library.sh.cn", and endpoint label is "cbdb". Therefore, we can add these format links to any place with "<a>" tag of web page where we need to get serialization formats of this resource as shown in Fig 7. And the screenshot of "Li Qingzhao" page is shown in Fig 8, where the metadata formats section gives a variety of serialization format links. The details page for each person in the CBDB_LD system contains this section using the LDPS service.

#### 2. LDKS service

The LDKS service is used in the CBDB_LD project to display the linking information between different knowledge bases. In this example, the resource "Li Qingzhao" has linked to "Shanghai Library Name Authority File" and "VIAF", so we can call the LDKS service to describe relationship between them.

**Fig 9:**
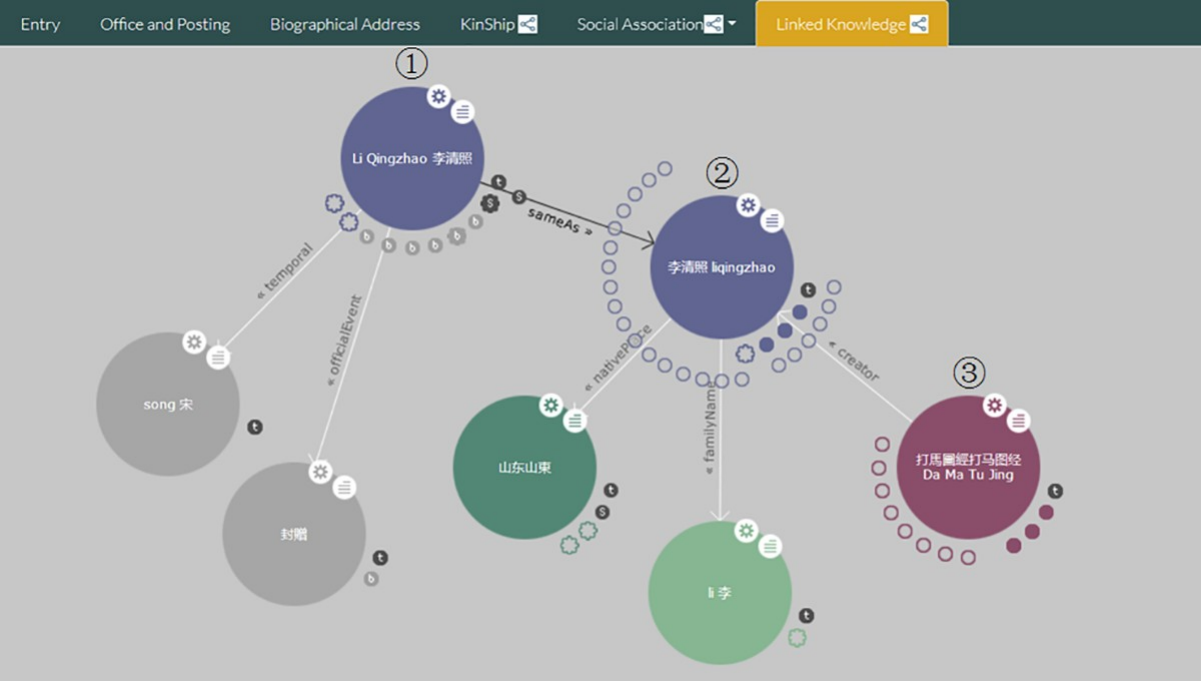
LDKS service for resource "Li Qingzhao".

When accessing each person's page, the knowledge graph of Fig 9 is included, and this graph is automatically generated based on the connected resources of different datasets. As shown in Fig 9, this knowledge graph contains three datasets as follows.

1. Node 1 is drawn from CBDB_LD knowledge base. There have some information extracted from this base in this node, such as temporal and officialEvent.
2. Node 2 is drawn from "Shanghai Library Name Authority File" dataset which contains nativePlace and familyName data. Node 1 and node 2 are connected with property "owl:sameAs".
3. Node 3 is a work existed in "Chinese Ancient Books Union Catalogue and Evidence-based Platform" which is connected to node 2 with property "bf:creator".

**Fig 10:**
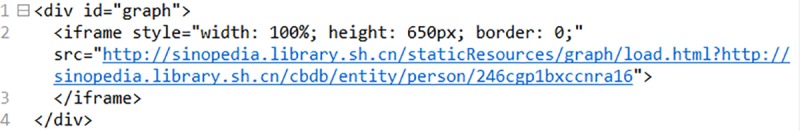
Code for LDKS service in a program.

If you want to use the LDKS service of SinoPedia, the following code in Fig 10 with "<iframe>" tag should be inserted in web page, and you can draw the same knowledge graph like Fig 9.

### Case 2: Digital Chorography Integration Platform (DCIP)

**Fig 11:**
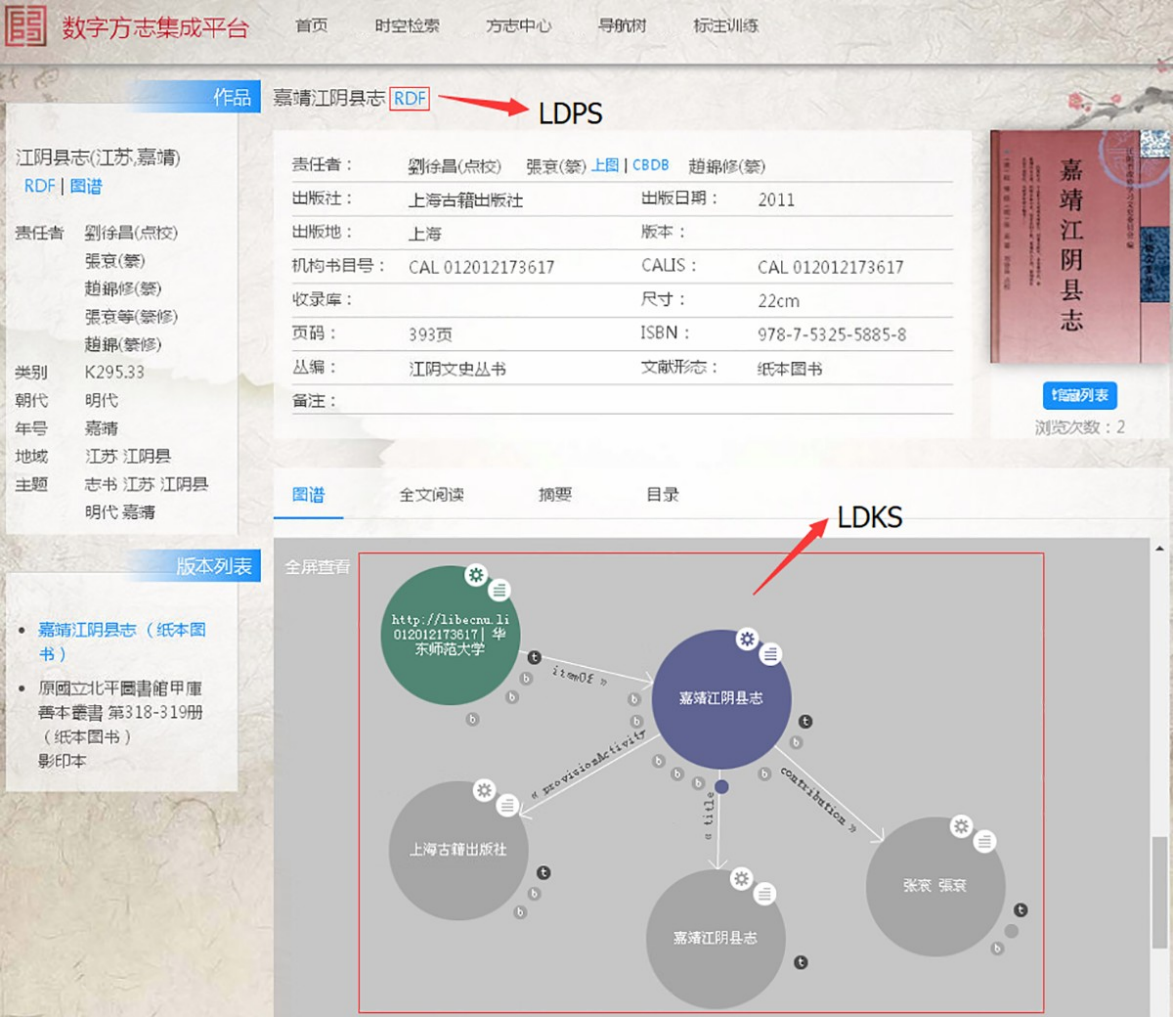
Applied LDPS & LDKS in ECNU DCIP application.

This DCIP is built by digital humanities team of East China Normal University (ECNU) which also applies the linked data services by LDPS platform. This platform has more than 60 thousands bibliography works, which mainly uses bibframe 2.0, foaf and Shanghai Library temporal ontologies for knowledge organization. The raw data for these bibliography works is MARC format stored in relational database. Therefore, we firstly need to convert MARC format into RDF format. In the presentation of chorography work and its instances, the LDPS and LDKS services are also used which are shown in Fig 11. The RDF/HTML format interface is used in LDPS, and instance and external open datasets are connected in LDKS.

**Fig 12:**
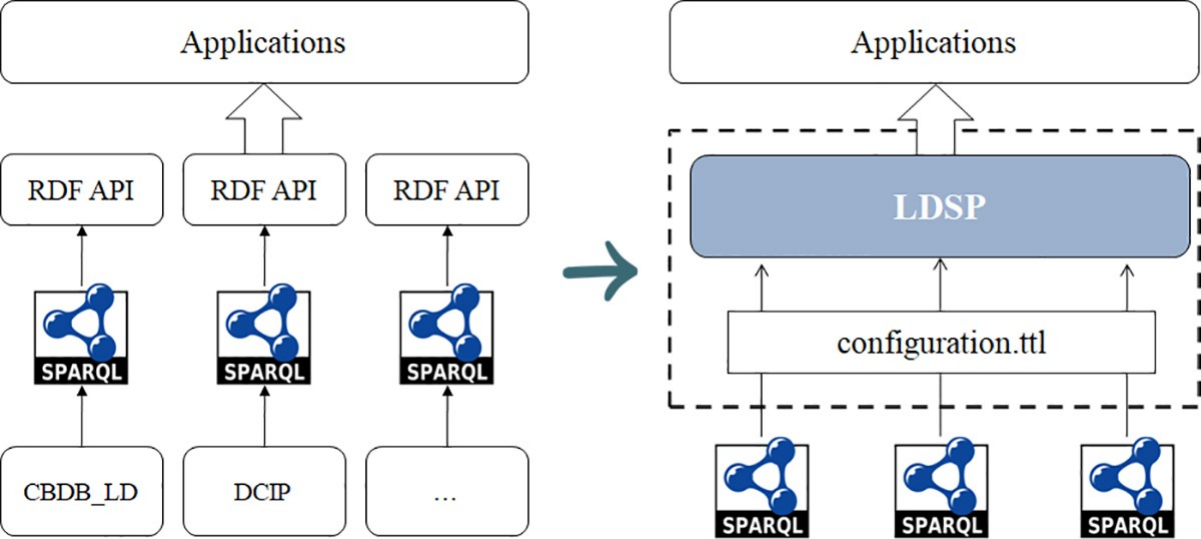
Traditional publishing method vs. LDSP.

Finally, let’s compare the difference between traditional publishing method for RDF data and using LDSP platform as illustrated in Fig 12. In the traditional publishing process, the publishing platform or API interface needs to be embedded in each application, which is used to provide publishing services for other system calls. There are some disadvantages in this approach. For application publishers, it will cause update synchronization problems, because all applications will need to be updated when the publishing platform or interface is upgraded. For data consumers, they must remember the access address of each application to get RDF data for resources. However, with LDSP, publishers only need to provide their SPARQL endpoints, while consumers only need to access the LDSP address without considering these different data sources. In other words, LDSP will make it less difficult to publish and reuse RDF data.

## Conclusion

Through the above modeling and operation instructions, we presented the SinoPedia platform which offers a number of benefits over many other single knowledge bases. The principal benefit is the unified publishing mechanism for scattered endpoints using a simple configuration. This platform is also providing linked data services, including LDTS, LDQS, LDPS and LDKS. These services can be used in any application as long as the SPARQL endpoint is provided, e.g. data publishing, content negotiation, and knowledge graph.

According to these optimized methods, we will integrate another two services, Linked Data Reasoning Service (LDRS) and Linked Data Intelligence Service (LDIS). The LDRS will encapsulate some common reasoning tools and rules to provide easily reasoning between different knowledge bases. The LDIS aims to offer semantic computing based on the graph.

In summary, these six services will form all the features of the linked data application framework which will be reused by other linked data applications and systems with API interfaces.
